# Peripheral Blood Classical Monocytes and Plasma Interleukin 10 Are Associated to Neoadjuvant Chemotherapy Response in Breast Cancer Patients

**DOI:** 10.3389/fimmu.2020.01413

**Published:** 2020-07-09

**Authors:** Javier Valdés-Ferrada, Natalia Muñoz-Durango, Alejandra Pérez-Sepulveda, Sabrina Muñiz, Irenice Coronado-Arrázola, Francisco Acevedo, Jorge A. Soto, Susan M. Bueno, Cesar Sánchez, Alexis M. Kalergis

**Affiliations:** ^1^Instituto Milenio de Inmunología e Inmunoterapia, Departamento de Genética Molecular y Microbiología, Facultad de Ciencias Biológicas, Pontificia Universidad Católica de Chile, Santiago, Chile; ^2^Departamento de Hematología-Oncología, Facultad de Medicina, Pontificia Universidad Católica de Chile, Santiago, Chile; ^3^Instituto Milenio de Inmunología e Inmunoterapia, Departamento de Endocrinología, Facultad de Medicina, Pontificia Universidad Católica de Chile, Santiago, Chile

**Keywords:** breast cancer, monocytes, cytokines, neoadjuvant chemotherapy, prognosis

## Abstract

Worldwide, breast cancer (BC) is the leading cause of cancer death among women. For many patients the most effective treatment is a resection surgery that removes the tumor. Within this subset, patients sometimes receive chemotherapy treatment (CT) prior to surgery aiming to reduce tumor size in order to preserve healthy breast tissue. This strategy is commonly called neoadjuvant chemotherapy (NAC). This approach also offers an opportunity to determine treatment sensitivity, especially in aggressive tumors. Post NAC absence of residual disease is associated to long term survival in BC patients and is used to define the need of adjuvant therapy options. Studies suggest that NAC allows the recognition of tumor antigens by immune cells potentiating the eradication of the tumor. However, the dynamic changes in patients' immune cells under NAC remain unclear. Here, we assessed changes in leucocyte and cytokine profiles in order to determine its association to NAC response in BC patients. Peripheral blood patient samples were taken prior to each NAC cycle to assess the abundance of leukocyte subsets and serum cytokines in 20 patients. These immunological features were associated with clinical outcomes including pathological response. We found a positive correlation between plasma Interleukin 10 (IL-10) and classical monocytes in HER2+ BC patients under NAC. We also observed a trend between increased IL-10 and classical monocytes levels and lower rates of pathologic complete response at the end of NAC. These data support the notion that monocyte subsets and IL-10 could be applied as a novel indicator of NAC efficacy in HER2+ BC patients. Finally, we confirm a key role of the immune system in cancer progression and CT response.

## Introduction

Currently, breast cancer (BC) is the most common cancer among women and the second most common overall. Only in 2018, a total of >2 million new cases were diagnosed (1). As occurs with other malignancies, BC is highly heterogeneous; BC subtypes include estrogen receptor (ER) positive (often called Luminal), human epidermal growth factor receptor type-2 (HER2)-enriched and triple negative breast cancer (TNBC). Some subtypes correlate with clinical and pathological characteristics, for example TNBC and HER2-enriched are more sensitive to chemotherapy (CT). To date, surgery is the most effective BC treatment. Sometimes, patients receive CT prior to surgery with the purpose of reducing tumor size in order to preserve healthy breast tissue. This strategy is called neoadjuvant chemotherapy (NAC). In this setting, pathologic complete response (pCR) is a useful clinical biomarker related to overall survival (2).

The immune system plays a pivotal role in cancer development and progression. Recently the widespread use of immune checkpoint modulators has revolutionize treatment for several solid neoplasms (3). In fact, Atezolizumab (an anti PDL-1 antibody) and Pembrolizumab (Anti PD-1 antibody) have been successfully used in TNBC, improving overall survival but only in the subgroup of tumors expressing high levels of programmed cell death ligand 1 (PD-L1–stained tumor-infiltrating immune cells of any intensity covering ≥1% of the tumor area), as determined by a U.S. Food and Drug Administration (FDA)-approved test (4), and pCR in the neoadjuvant setting; respectively (5). Specifically, clinical follow up of the PD-L1-positive subgroup treated with atezolizumab displayed significantly higher median progression-free survival (7.5 months) than did the PD-L1-positive subgroup treated with placebo, who displayed lower median progression-free survival (5.0 months) (4).

Inflammation favors tumor growth, metastatic spread and progression (6), it also increases oxidative stress and cell damage, leading to genomic alterations, aberrant gene expression and mutations (7). Tumor cells may also express molecules, such as surface calreticulin, tumor antigens or NKG2D ligands (8). These molecules are recognized by CD8^+^ effector cells and NK cells that target and destroy tumor cells (9). In a previous study, our group suggested that high neutrophil/lymphocyte ratio (NLR) in complete blood counts (CBC) could act as a predictor of death in BC patients (10).

Immune myeloid cells, such as macrophages, myeloid-derived suppressor cells (MDSC), and monocytes have been recently shown to play a major role in BC (11). Studies have shown increases in circulating MDSC in BC patient undergoing NAC treatment (12). Also, taxanes, alquilants (one of the main drugs using in NAC) may promote the generation of anti-tumorigenic human macrophages *in vitro* (13). However, the role of monocytes in BC is still unclear.

On the other hand, inflammatory markers, such as cytokines, play a crucial role for tumor progression. In particular, IFNγ and IL-10 have opposite roles in cancer (14). High IFNγ levels are associated to anti-tumor responses and a pro-inflammatory microenvironment (15). Activated CD8^+^ T cells release IFNγ inhibiting tumor cell proliferation and angiogenesis (15). Conversely, IL-10 contributes to tumor growth by inducing an immune response and an anti-inflammatory microenvironment that promotes tumor escape from immune surveillance (16). In general, increased IL-10 levels are associated to poorer prognosis, however its precise role in BC progression remains not fully understood (17).

Herein, we sought to correlate changes in leukocyte population subsets and cytokines levels in BC patients receiving NAC with pCR rates. We hypothesized that monocyte subsets and IL-10 could serve as predictive markers for pCR in these patients.

## Methods

### Study Goal

The Millennium Institute of Immunology and Immunotherapy (MIII) in collaboration with the Cancer Center of the *Pontificia Universidad Católica de Chile* and the *Complejo Asistencial Doctor Sótero del R*í*o* leaded the currently work. The main aim of the study was to evaluate whether the changes in population subsets of leucocytes in peripheral blood samples from patients with BC under NAC treatment could be useful as indicator of the efficacy of such treatment.

### Patient Recruitment, Chemotherapy Treatment, and Study Design

**Figure 1:**
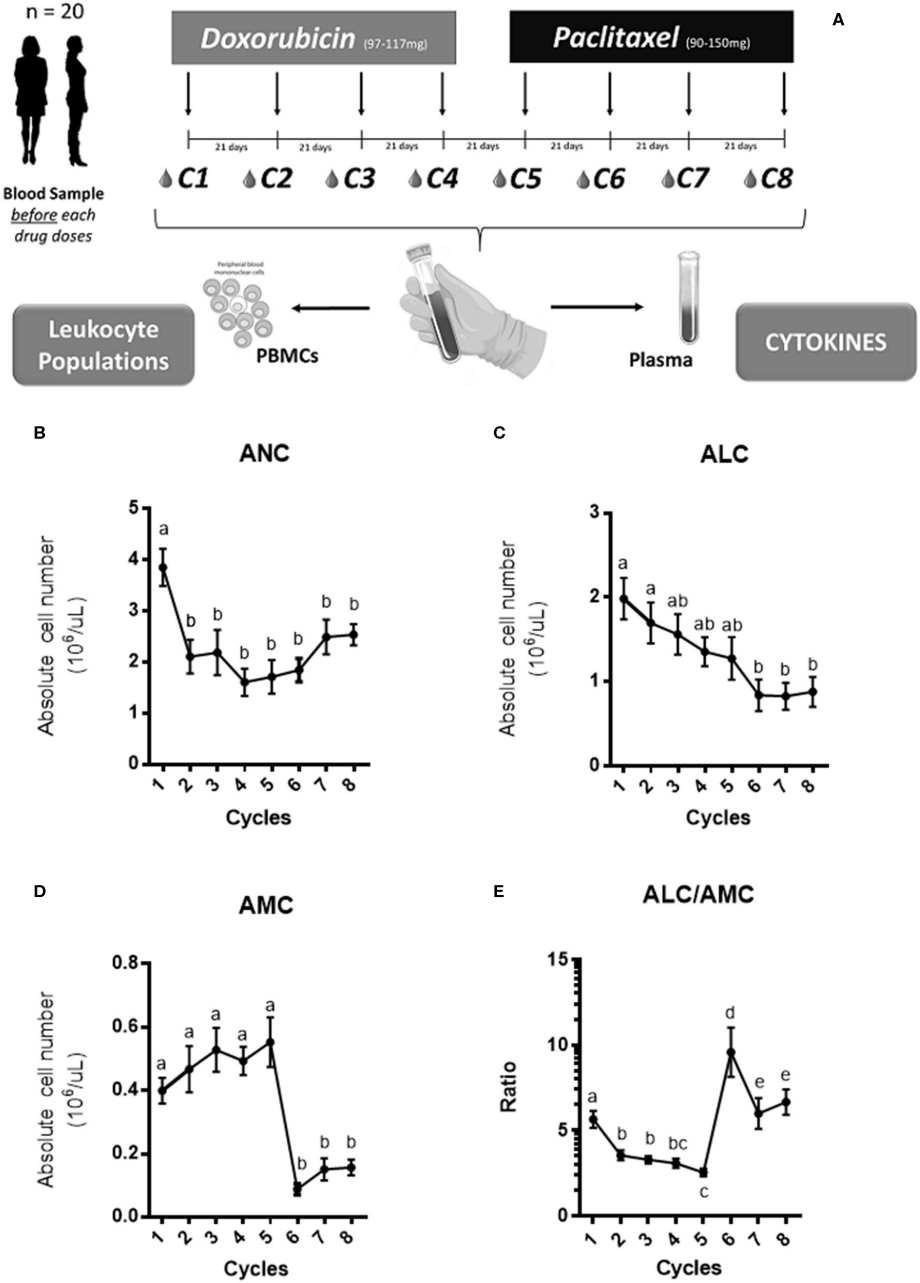
The experimental design and the hemogram analysis. Twenty patients received an intravenous NAC consisted in 4 cycles of doxorubicin at (97–117 mg) with cyclophosphamide at (910–1,175 mg) given every 21 days followed by four cycles of paclitaxel at (90–150 mg; twelve-doses weekly) given every 21 days. Blood samples were collected before the first dose of the drug in each cycle **(A)**. Both peripheral blood mononuclear cells (PBMC) and plasma were obtained to measure leukocyte cells and cytokines levels, respectively. The absolute neutrophil count (ANC), absolute lymphocyte count (ALC) and absolute monocyte count (AMC) across the NAC is shown in **(B–D)**, respectively. The ratio between both subsets ALC/AMC across the NAC is shown in **(E)**. The concentration for each cell group was expressed in 10^6^/μl and the mean with SEM was displayed. The statistical significances were assessed using a One-way ANOVA test and the significant differences were grouped in letters.

Adult (>18-year-old) female BC patients selected for NAC treatment were enrolled into this study. Intravenous NAC protocol consisted of four cycles of doxorubicin (DOX - 60 mg/m^2^) plus cyclophosphamide (600 mg/m^2^) every 21 days, followed by 12 doses of paclitaxel (PXL 90 mg/m^2^) weekly (3 doses of PLX (21 days) corresponding to one cycle). Additionally, HER2-enriched BC patients received trastuzumab (600 mg) every 3 weeks for 1 year. Blood samples were collected prior to the first DOX dose of each cycle and prior to the first PXL dose of each cycle. Blood samples were used to perform hemograms obtain peripheral blood mononuclear cells (PBMC) and serum. A total of eight time points were evaluated throughout the NAC treatment (Figure 1A).

### Purification of Peripheral Blood Mononuclear Cells From Blood Samples

Blood samples were collected in EDTA-tubes; PBMC were extracted with lymphocyte separation medium (LSM) according to the manufacturer's protocol (Corning, 25-072-CV, Virginia, USA). Viable PBMC were counted in Neubauer chamber using Trypan Blue staining. Purified PBMCs were frozen in 10%FBS-DMSO. Vials were stored at −80°C overnight and then transferred into −150°C until used.

### Flow Cytometry Analyses

To evaluate the leukocyte populations, 5 × 10^5^ cells of the thawed PBMCs were seeded in 96-well plates (Additional file 1: Figure S1) and stained with a panel of humans antibodies CD4-PE (Clone, RPA-T4 BD bioscience), CD8-BB515 (Clone, RPA-T8 BD bioscience), CD3-PeCy7 (Clone, SK7 BD bioscience), CD14-BV510 (Clone, M0P9 BD bioscience), CD16-APC (Clone, 3G8 BD bioscience) and CD56-BUV-395 (Clone, NCAM16.2 BD bioscience), according to manufacturer instructions. Cells were resuspended in 100 ul of PBS 1%FBS containing the mix of antibodies and incubated at 4°C for 30 min. After incubation, cells were washed twice with PBS and centrifuged at 400 g for 5 min at 4°C. Then, the cell pellet was resuspended in 200 ul of PBS 1%FBS. Samples were acquired at the LSRFortessa X20 flow cytometer (BD Biosciences) and analyzed using FlowJo v10.0.7 software (BD Biosciences). Gating strategy used to identify lymphoid and myeloid subsets is showed in additional file 1: Figure S1.

### Plasma Extraction and Cytokines Measurement From Blood Samples

Blood samples were centrifuged at 2,500 rpm at room temperature for 15 min. Then, plasma was removed, and 2 ml aliquots were frozen at −80°C. Plasma cytokines were quantified using the OptEIA™ human IFNγ and IL-10 kits (Biosciences BD) according to the manufacturer's protocol.

### Assessment of Clinical Responses

Biopsies were obtained at the beginning of the study to confirm BC diagnosis and to identify tumor subtype (2, 18). Also, at the end of NAC (8th cycle) all patients were subjected to tumor resection and histopathological analyses were performed to evaluate the pathologic response defined as a total, partial or not-response. The histopathological studies were performed by an expert clinician under blind methodology.

### *In vitro* PBMC Stimulation Assay

To evaluate the cytokine profile across the NAC treatment in BC patients, 500,000 PBMCs/well (previously thawed) were seeded and stimulated with 5 μg/ml of αCD3/CD28, 2.5 μg/ml of concanavalin A (ConA) or 0.5 μg/ml of lipopolysaccharide (LPS). Unstimulated cells were included as negative controls. The cells were incubated for 48 h at 37°C 5% CO_2_. After the incubation, the cells were separated, and supernatant was collected for the future assay. Afterwards, the levels of IFN-γ and IL-10 cytokines of these samples were measured by ELISA according to manufacturing protocol (Biosciences BD IFNγ, and IL-10 BD OptEIA™ human kits).

### Statistical Analyses

Statistical analyses were performed in GraphPad v.6.0. Statistical significance was assessed by One-way or Two-way ANOVA followed by Tukey's post-test. Spearman correlation was assessed to study the relationships among BC types and cell subsets. Finally, a contingency table analyzed Chi-square or Fisher's test for the pCR data. Statistical significance was set at *p* < 0.05 and r values in a range between −1 and 1.

## Results

### Patient Characteristics

A total of 20 BC patients were recruited into this study. Clinical characteristics are summarized in Table 1. In line with previous reports in the Chilean population median age was 48 years (range: 30–62) (19). Median weight and body mass index (BMI) were 79 kg and 29.5; respectively. Regarding BC subtypes, the most frequent was ER+ (*n* = 13) followed by HER2 enriched (*n* = 5) and TNBC (*n* = 2). Confirming the subtype distribution previously reported by our research group (20, 21).

**Table 1:** Clinical characteristics of patients.

**Characteristics**	***N* (%)**
Total of patients	20
Age (years)	
Median	46
Range	30–62
Weight (kg)	
Median	79
Range	58–100
BMI (kg/m^2^)	
Median	29.5
Range	20–39
Tumor HER2	
Positive	14 (70)
Negative	6 (30)
BC Subtype	
ER+	13 (65)
HER2-enriched	5 (25)
TN	2 (10)

*BMI, Body Mass Index; HER2, Human Epidermal growth factor type 2 receptor; BC, Breast cancer; ER+, Estrogen Receptor positive; TN, Triple Negative*.

### BC Patients Under NAC Showed Decreasing Leukocyte Counts

As shown in Figure 1A, NAC was associated to a reduction of leukocyte counts in all patients. Then, to assess the impact of CT in specific leukocyte subpopulations we quantified absolute neutrophil count (ANC), the absolute lymphocyte count (ALC) and absolute monocyte count (AMC) from CBC. We found that ANC and ALC decreased significantly along the NAC (Figures 1A,B). On the other hand, AMC levels increased during the first cycles of NAC and decreased after the 5th cycle (Figure 1C). Finally, we calculated the monocyte/lymphocyte ratio; Figure 1D shows an increase in AMC/ALC during the first NAC cycles followed by a drastic decrease after the 5th cycle, concomitant to the switch in chemotherapeutic drug. Both lymphopenia (22) and neutropenia (23) are commonly seen in patients under CT treatment and could explain this decrease.

### Across NAC Cycles, Classical and Non-classical Monocytes Subsets Show a Dynamic Change in Their Proportions

**Figure 2:**
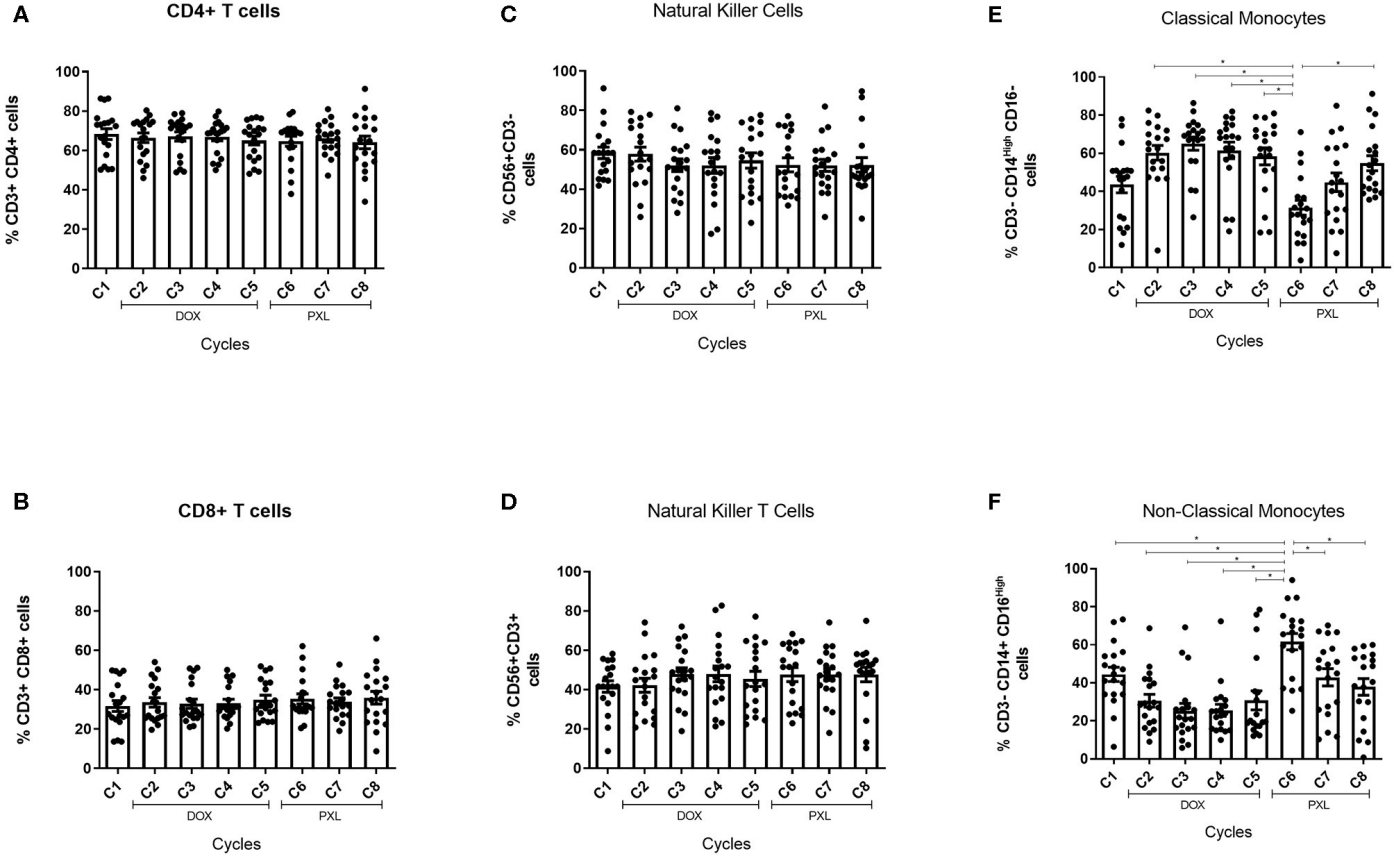
Peripheral blood mononuclear cells characterization under NAC in BC patients. Cells were obtained from the PBMC previously extracted from blood samples of BC patients (*n* = 20) and the characterization were performed by flow cytometry. The percentage of CD4^+^ and CD8^+^ cells was calculated from the CD3^+^ and showed across the NAC treatment in **(A,B)**, respectively. The percentage of NK cells was calculated from the CD56^+^ and CD3^Neg^ and the data was showed across the NAC in **(C)**. Also, the percentage of NKT cells was calculated from the CD56^+^ and CD3^+^ and was showed in **(D)**. The percentage of classical monocytes was calculated from the CD14^High^ CD16^Neg^ gated in CD3^Neg^ **(E)** and the non-classical monocytes was calculated from the CD14^+^ CD16^High^ percentage **(F)**. The cycle 1 (C1) was the percentage of cells without the effect of the treatment (baseline). The statistical significances were assessed using a One-way ANOVA test *p < 0.05.

In order to assess changes in mononuclear cell populations associated to NAC we performed flow cytometry analyses. First, we focused on the CD4^+^ and CD8^+^ T lymphocytes along with NK (CD3^Neg^ CD56^+^) and NKT (CD3^+^ CD56^+^) cells (8, 14, 24). Then, to understand the potential contribution of monocytes during NAC we classified these cells as classical (CD3^Neg^ CD14^High^ CD16^Neg^) or non-classical (CD3^Neg^ CD14^+^ CD16^High^) monocytes according to surface markers (25, 26). Neither CD4 nor CD8 T cells displayed significant differences in their percentages across NAC (Figures 2A,B). Similar results were obtained for NK (CD3^Neg^ CD56^+^) and NKT cells (CD3^+^ CD56^+^) (Figures 2C,D). Classical monocytes (CD3^Neg^ CD14^High^ CD16^Neg^) increased significantly after the first cycle of NAC with DOX and remained high. After the PXL treatment was started (6th cycle), classical monocytes decreased but the percentage of these cells was later restored. Importantly, non-classical monocytes (CD3^Neg^ CD14^+^ CD16^High^) significantly decreased after the first cycle of NAC and tended to increase during the 6th cycle (Figure 2E), displaying an opposite pattern compared to classical monocytes (Figure 2F). In summary, flow cytometry analyses confirmed hemogram results shown in Figure 1, demonstrating dynamic changes in monocyte populations by NAC. According to our data, these increased numbers of AMC in peripheral blood could be due to classical monocytes (CD3^Neg^ CD14^High^ CD16^Neg^).

### IL-10 and IFNγ Plasma Levels Increase During NAC in BC Patients

**Figure 3:**
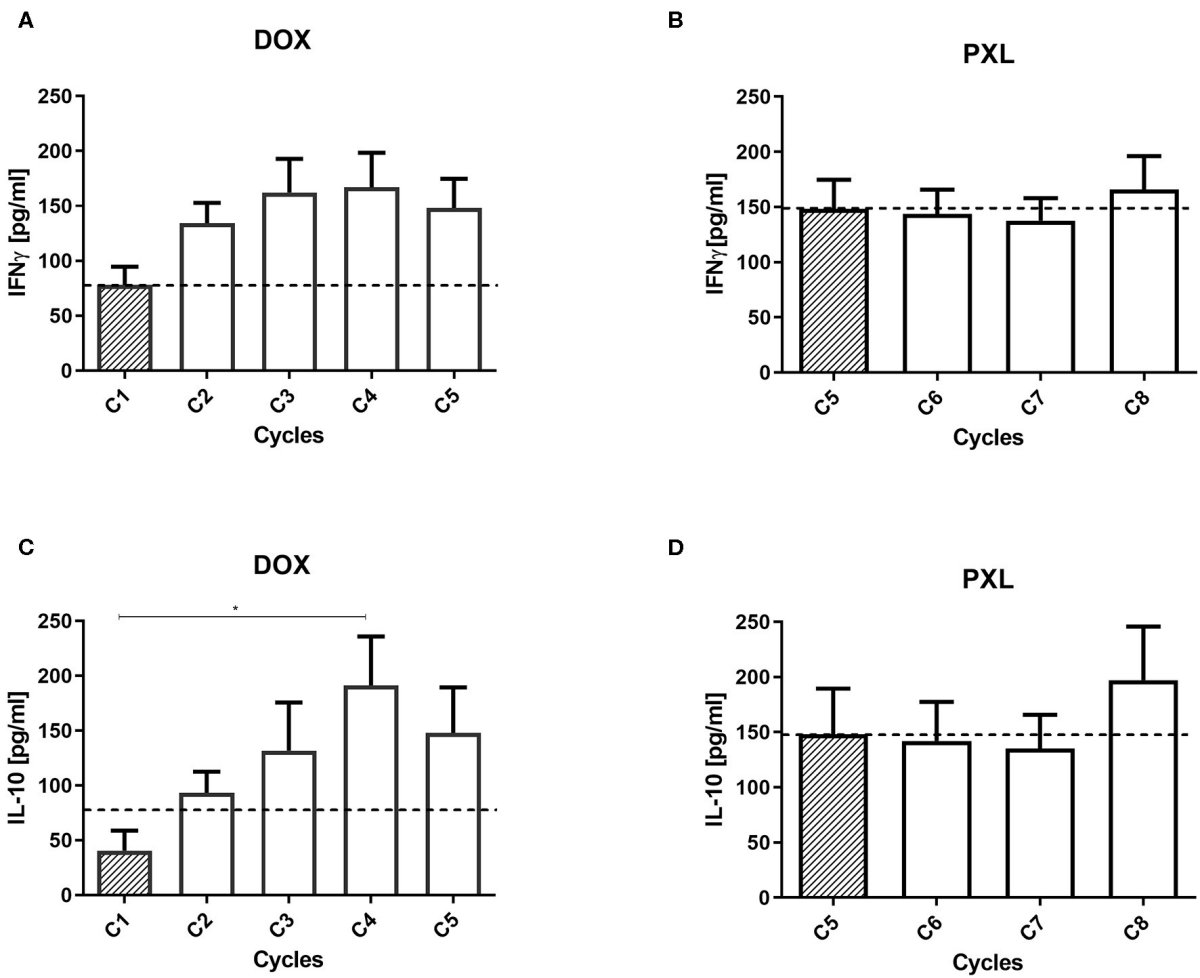
IFNγ and IL-10 levels in plasma from breast cancer patient. The cytokines were measured in plasma samples from BC patients across the NAC cycles (*n* = 20). For both cytokines the cycle 1 (C1) is the effect of the treatment without any drug (baseline). In **(A)** the effect of the doxorubicin (DOX) for IFNγ and in **(B)** for paclitaxel (PLX). Also, in **(C)** the effect of DOX and in **(D)** the effect of PLX for the IL-10 plasma levels. Statistical significances were assessed using a One-way ANOVA test *p < 0.05.

In addition to peripheral leukocytes, we measured serum cytokines during NAC. While IFNγ plasma levels showed a tendency to increase (*p* = 0.1333) during the NAC/DOX treatment (Figure 3A), the levels of this cytokine remained stable during PLX treatment (Figure 3B). Similarly, IL-10 levels significantly increased during DOX treatment between the first and third NAC cycle (*p* = 0.0332; Figure 3C); IL-10 levels also remained stable during PLX treatment (Figure 3D). We also measured the plasma levels of IL-12 (non-detected), TNF-α and TGF-β (Additional file 2: Figure S2), but we did not see any tendency or secretion profile during NAC.

### Percentage of Classical Monocytes Decreased During NAC and Non-classical Monocytes Increased in HER2-Enriched and Luminal Breast Cancers

**Figure 4:**
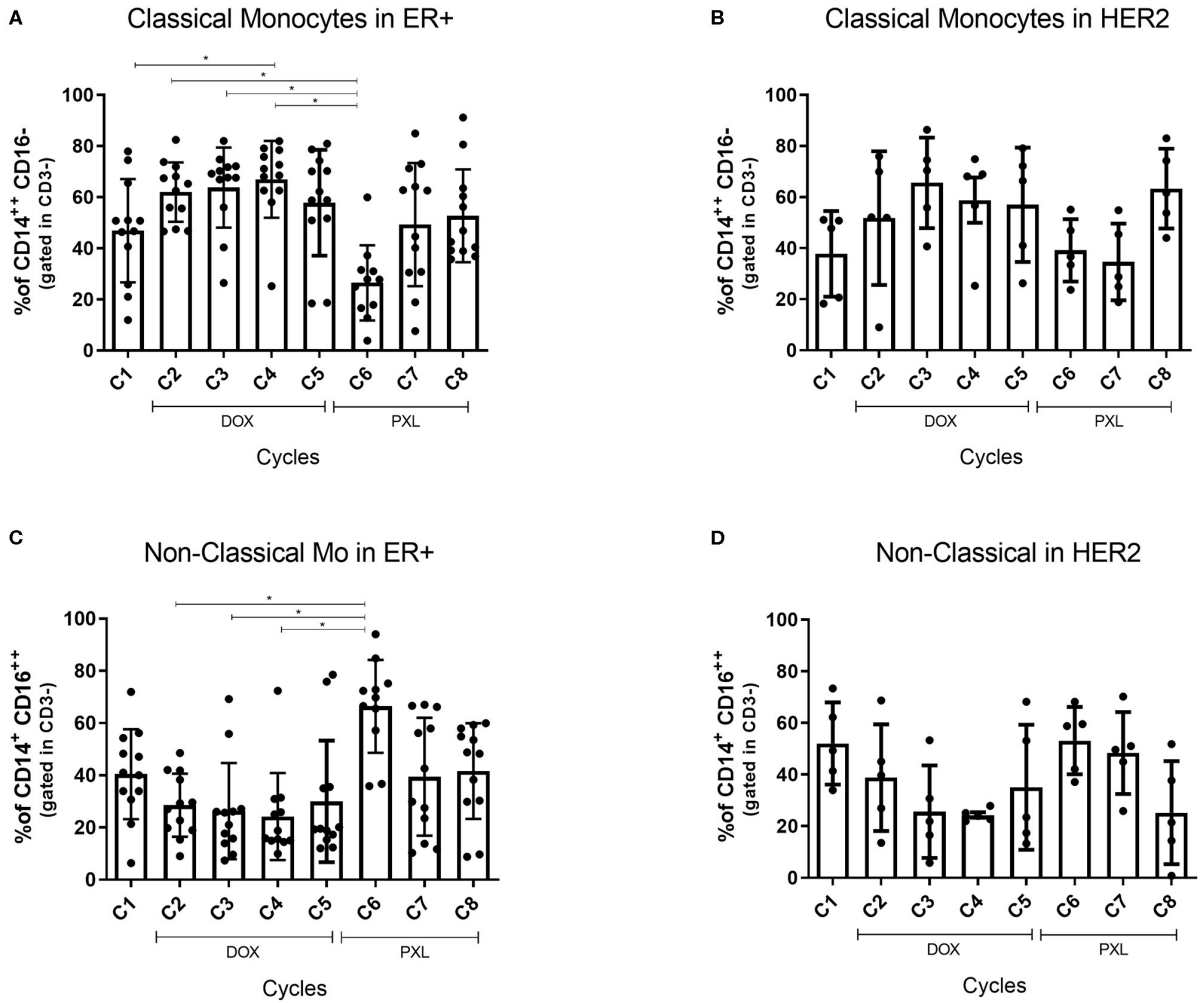
Classical Monocytes and Non-Classical Monocytes percentage by breast cancer subtype. Monocytes cells were obtained from the PBMC previously extracted from blood samples of BC patients (*n* = 20). The percentage of monocytes cells was calculated by flow cytometry from the CD14^High^CD16^Neg^ gated in CD3^Neg^ for classical monocytes and CD14^+^CD16^High^ gated in CD3^Neg^ for the non-classical monocytes. This data is shown across the NAC cycles. The Classical Monocytes distributed in ER+ cancer and HER2+ is shown in **(A,B)**, respectively. The same distribution for non-classical monocytes is shown in **(C,D)**. The cycle 1 (C1) was the percentage of cells without the effect of the treatment (baseline). The statistical significances were assessed using a One-way ANOVA test *p < 0.05.

Since monocyte subsets were the only population that showed significant changes by NAC treatment (Figure 1), we analyzed the distribution of these cells by BC subtypes. We grouped the percentage of classical or non-classical monocytes by ER+ and HER2-enriched BC (Figure 4). TNBC subtype was excluded from this analysis given the low number of patients (*n* = 2). Importantly, the percentage of classical monocytes cells (CD3^Neg^ CD14^High^ CD16^Neg^) increased during treatment with DOX and decreased with PLX, showing a significant difference compared with the 6th NAC cycle in ER+ BC patients (Figure 4A). Nevertheless, classical monocytes in HER2+ BC patients only showed a tendency to increase across cycles (Figure 4B). On the other hand, non-classical monocytes (CD3^Neg^ CD14^+^ CD16^High^) tended to decrease with DOX and to increase with PLX in both subtypes showing significant difference against the 6th NAC cycle in the ER+ BC subtype (Figure 4C). In summary, significant differences between cycles were only found in the luminal group for both monocyte subsets. In addition, an opposite distribution was showed for classical and non-classical monocytes across the NAC (Figures 4A,C).

### Increased Levels of IL-10 Correlate With Increased Percentage of Non-classical Monocytes in HER2 Enriched Breast Cancer

**Figure 5:**
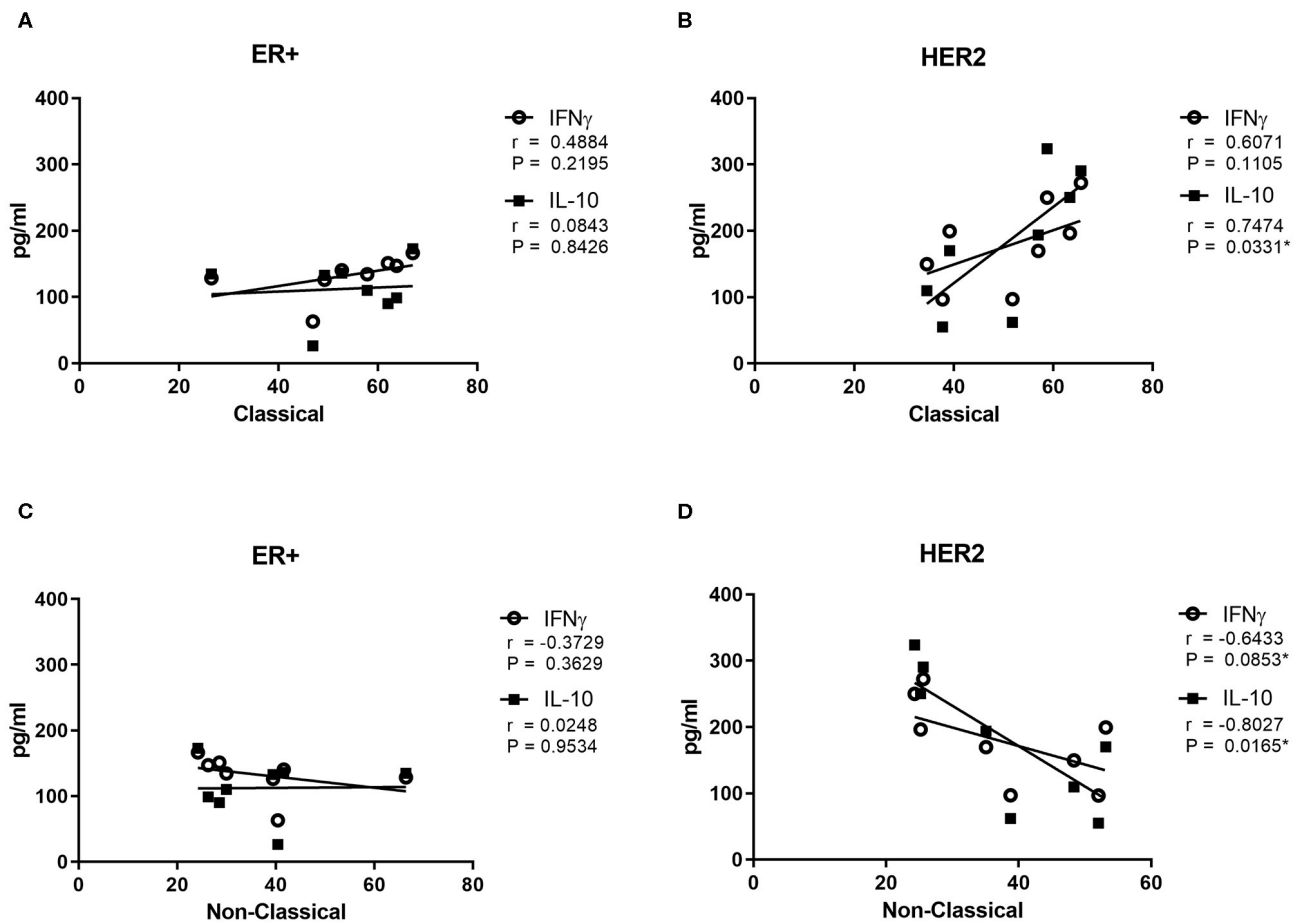
Classical and Non-Classical Monocytes correlation between cytokines levels and breast cancer subtype. Cytokines levels in plasma were measured in the blood samples of patient with breast cancer previously extracted (*n* = 20). The results were grouped by kind of BC and monocytes subset. In squares are displayed the IL-10 levels and in circles the IFNγ levels. Cytokines levels are presented against the media percentage of each monocyte's subset. The classical monocytes data is divided in ER+ and HER2+ BC and it is exhibited in **(A,B)**, respectively. The same distribution for non-classical monocytes is shown in **(C,D)**. The Spearman analysis was used to study the relationships among BC types, cytokines levels and monocytes subsets. The differences were considered significant when *p* < 0.05.

Next, correlation analyses between IFNγ or IL-10 plasma levels and the percentage of non-classical and classical monocytes were performed across the NAC and the ER+ or HER2-enriched BC (Figure 5). No significant correlations in ER+ BC for cytokine levels and monocyte subsets were observed (*p* = 0.2195 for IFNγ and *p* = 0.8426 for IL-10). However, the IFNγ levels showed a tendency to a positive Spearman coefficient in classical monocytes (*r* = 0.4884) and a negative one (*r* = −0.3729) in non-classical monocytes (Figures 5A,C, respectively). Remarkably, the IFNγ levels in HER2-enriched BC did not show a significant correlation for any monocyte subset (*p* = 0.1105 for Figure 5B and *p* = 0.0853 for Figure 5D). However, the highest Spearman coefficient (*r* = 0.6071) for classical monocytes suggests a correlation between increasing levels of IFNγ and high percentage of classical monocytes (Figure 5B). In addition, an inverse correlation was found between non-classical monocytes and IFNγ levels (Figure 5D) (*r* = −0.6433). On the other hand, the cytokine levels grouped by monocytes subset in HER2-enriched BC showed a significant correlation between IL-10 levels for both classical monocytes and non-classical monocytes (*p* = 0.0331 and *p* = 0.0161, respectively) (Figures 5B,D). The highest levels of IL-10 were correlated with high percentages of classical monocytes with a positive Spearman coefficient (*r* = 0.7474) (Figure 5B). On the other hand, an opposite correlation is showed for non-classical monocytes and the IL-10 levels in HER2-enriched patients. The lowest levels of IL-10 were correlated with high percentage of non-classical monocytes and a negative Spearman coefficient (*r* = −0.8027) (Figure 5D). In summary, only for HER2-enriched patients and IL-10 levels we saw a significant difference between monocytes subsets.

**Figure 6:**
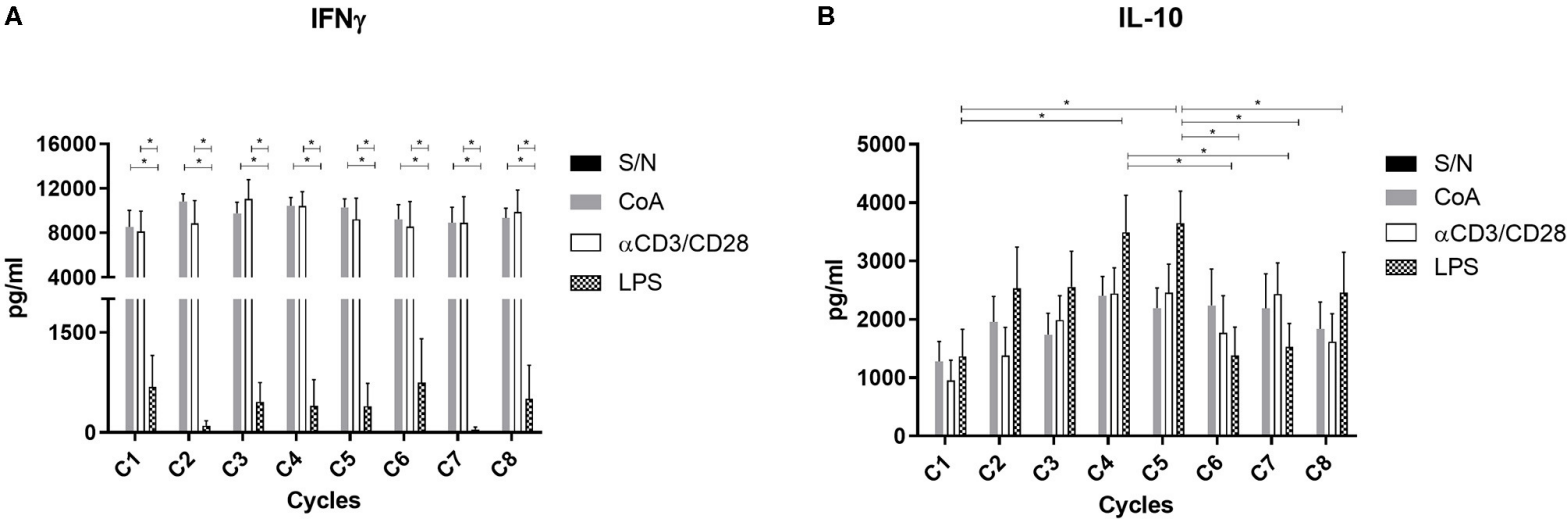
Stimulation assay and cytokine levels across the NAC. The PBMC previously extracted from blood samples in BC patients were stimulated with ConA (2.5 μg/ml), αCD3/CD28 (5 μg/ml), LPS (0.5 μg/ml) or without stimulus for 48 h in a CO_2_ incubator at 37°C. After incubation IFN-γ **(A)** and IL-10 **(B)** levels were evaluated by ELISA. Two-way ANOVA test and *p < 0.05.

To explore the potential source of IFN-γ and IL-10, PBMC were stimulated *in vitro* with three different molecules, each of them to promote cytokine production from lymphoid (Concanavalin A or anti-CD3/CD28) or myeloid cells (LPS) (27, 28). The main source of IFN-γ came from these cells stimulated with Concanavalin A or anti-CD3/CD28, indicating that the source was from lymphoid cells (Figure 6A). In contrast, the levels of IFN-γ obtained from myeloid cells stimulated with LPS was significantly lower (Figure 6A). On the other hand, secretion of IL-10 was induced with all stimuli used (Figure 6B); however, the main source of IL-10 secretion came from myeloid cells that respond to LPS stimulation (Figure 6B). In addition, only cell stimulated with LPS displayed a significative increasing IL-10 production across NAC cycles, displayed the higher level at cycles 4 and 5 (Figure 6B). After cycle 5 only cells stimulated with LPS decreased the levels of IL-10 secretion, in contrast with levels of IL-10 secreted from lymphocytes stimulated with Concanavalin A and anti-CD3/CD28. Above results suggest that main source of IL-10 during NAC coming from myeloid cells.

### IL-10 Levels and Classical Monocytes From a Pathologic Complete Response May Be Used as a Predictive Indicator in Breast Cancer Patients

To associate the changes observed during the NAC in monocyte populations, IL-10 and IFNγ with the clinical outcome, we calculated the proportion of pCR based in the histological analyses of biopsies obtained from definitive surgery of the primary tumor. Results were classified as total responder for those patient biopsies that displayed no residual invasive tumor, partial responder for those patient biopsies with a moderate response to the NAC, and no response in patients without an effect of the NAC in the biopsies. We built contingency tables for pathological response and plasma cytokines (Table 2), and the peripheral blood monocytes (Table 3). We divided the cytokine levels and the monocyte subsets percentage in low and high. These parameters were determined by the basal levels observed in the first cycle of NAC. Unfortunately, we did not obtain enough blood sample in the last NAC cycle for each patient to perform both analyses, therefore our total of patients decreased from 20 to 15 in Table 2 and from 20 to 19 in Table 3. Both tables contain the number of patients for each category previously described. We perform a Fisher's test to evaluate the association between the cytokine levels and the pathological response. We found that highest levels of IL-10 were associated with a partial response, but without a statistically significant results (*p* = 0.1357 for IL-10 and *p* = 0.3287 for IFN-γ). Furthermore, the relative risk for IL-10 was 3 (95% CI 0.7799–11.540) and 2 for IFNγ (95% CI 0.6102–6.555). For the monocytes subsets analyses, we perform a Chi-square test. In the same way, a highest percentage of classical monocytes showed to be more frequent in patients with partial response. However, there was no statistically significant difference between the subset's percentage and the pCR (*p* = 0.2690 for classical monocytes and *p* = 0.5174 for non-classical monocytes).

**Table 2:** Plasma cytokine levels vs. complete pathologic response.

**Plasma cytokines**	**Biopsies 8th cycle results**	**Low production 0–100 pg/ml**	**High production >100 pg/ml**	**Fisher's test (α** **=** **0.05)**	
				***P-*value**	**Relative risk**
IL-10	Total response	4	2	0.1357	3 (0.7799–11.540)
	Partial response	2	7		
IFNy	Total response	3	2	0.3287	2 (0.6102–6.555)
	Partial response	3	7		

*Low and high levels of cytokine production were determined by the basal levels observed before cycle 1*.

**Table 3:** Monocytes subsets levels against the complete pathologic response.

**Monocytes subsets**	**Biopsies 8th cycle results**	**Low percentage 0–40**	**High percentage >40**	***x***^****2****^ **test (α** **=** **0.05)**	
				***P-*value**	***x*^**2**^ value**
Classical	Total response	3	3	0.2690	2.626
	Partial response	1	7		
	No response	1	4		
Non-Classical	Total response	2	4	0.5174	1.318
	Partial response	5	3		
	No response	3	2		

*Low and high percentage of monocytes were determined by the basal levels observed before cycle 1*.

## Discussion

Inflammation (29) is widely recognized as a “hallmark of cancer” (30). Chronic systemic inflammation correlates with poorer outcomes in BC patients (31). Systemic markers, such as cytokines secretion or changes in leukocytes ratios could help to obtain a more accurate clinical follow up. These changes could also serve as prognostic factors in patients receiving CT (17, 31, 32).

In agreement with our results, a study in BC patients reported no differences in CD4^+^/CD8^+^ or NK cells by NAC (33). The ratio pf peripheral leukocytes have been used as prognostic factors for chemotherapeutic interventions (31). Previously, we and others have reported that the neutrophil-to lymphocyte ratio (NLR) could be useful as prognosis factor in BC confirming a role of myeloid cells in this malignancy (10). Lymphocyte to monocyte ratio (LMR) has also been explored as predictor for progression in BC patients under NAC (34–36). Accordingly, low LMR patients displayed shorter disease-free survival, suggesting a prognostic role of LMR. However, none of these studies found a significant correlation with pCR (35). Consequently, our study was not limited to peripheral monocytes counts prior and after NAC, it also included LMR and phenotypic changes in monocytes subsets during NAC.

In clinical studies the presence of CD14^+^CD16^+^ cells indicates inflammatory disease (37). These monocytes increase their levels in several human diseases such as atherosclerosis, rheumatoid arthritis and Crohn's disease (26). On the other hand, non-classical monocytes may have a patrolling role in vessel walls, infiltrating tissues following the interaction between CXCR1 and CCL3 (38). This subset can also secrete inflammatory cytokines such as TNF-α and IL-1β upon inflammatory stimulation (38). In our study the characterization of both types of monocytes displayed significant differences in the levels of non-classical/classical monocytes during NAC (Figures 2E,F). These differences peaked in the 6th NAC cycle, coinciding with the first measured point for the PLX drug suggesting both drugs could modulate the response of these cells. Although, we fail searching similar studies performed in human monocytes that explain this result, we found an *in vitro* study performed in murine dendritic cells (mDCs) treated with no-toxic doses of chemotherapeutics (39). Here it was shown that DOX and PLX enhance the antigen processing machinery and induced higher expression of surface maturation markers in DCs, suggesting an immunogenic phenotype. Also, these cells secreted higher levels of IL-12 in contrast to control (39). Other studies performed in human DCs derived from monocytes, showed that both DOX and PLX increased the levels of MHC-II and CD80 surface expression (40, 41). These studies indicate that both drugs can modulate the function of myeloid cells, however the specific mechanism in different subsets of monocytes must be explored in further studies.

We observed increased levels in the IFNγ and IL-10 secretion in DOX treatment of NAC (Figure 2), but only IL-10 showed a statistically significant difference between some cycles (Figure 2C). The PLX treatment showed stable levels of both cytokines in plasma. Those cytokines are described with contrary effects in the tumor carcinogenesis (15). The first one, IFNγ, is associated with an anti-tumor response and is an inflammatory marker in the elimination of the tumor (42). On the other hand, IL-10 has been classically associated with an immunosuppressive microenvironment and may promote tumor development and progression (24). Unfortunately, our results in plasma did not clarify an unbalance for both cytokines and they may be are not directly related with the effect of the CT in BC patient.

In order to establish a potential relationship between plasma cytokines and monocyte subsets we performed a Spearman correlation analysis. Our data also was analyzed by BC subtypes. Unfortunately, we did not find a significant correlation between ER+ BC and classical (Figure 5A) or non-classical monocytes (Figure 5C) monocytes. The highest percentage of classical monocytes was correlated with the highest IL-10 levels in HER2-enriched BC (Figure 5B). Previous reports demonstrate that classical monocytes may secrete IL-10 (43). In contrast, plasma IL-10 levels were lower when the percentage of non-classical monocytes decreased in HER2-enriched BC (Figure 5D). Although we could not perform intracellular IL-10 stain to decipher whether monocytes were the source of these cytokine; we performed *in vitro* stimulation with LPS which stimulates principally to monocytes (Figure 6) and dendritic cells (27). This result displayed that only cells stimulated with LPS induced significant high levels of IL-10 comparing first cycles of NAC to 5th cycle. This result is also in agreement with the time-lapse of monocytes increasing displayed in Figures 2E,F. On the other hand, it is highly described that various myeloid cells of the immune system are capable of secreting IL-10 such as DCs, macrophages and even neutrophils (44). An additional study demonstrated that plasmacytoid dendritic cells located in draining lymph nodes are an important source of cytokines in BC (45). They associated increased levels of IL-12 and IFNγ produced by PCD, with good prognostic, and IL-4 and IL-10 with poor prognostic. Therefore, our data suggest a relationship between classical monocytes and plasma IL-10 in HER2-enriched BC patients with PLX mediated the abundance of the monocytes' subsets.

Previous reports have described that PLX may induce maturation of mouse macrophages and dendritic cells via TLR4, mimicking the effects of LPS (46). We speculate that DOX induces differentiation of classical monocytes in HER2-enriched BC. These cells may secrete IL-10 (26). The second part of NAC with PLX may change the breast microenvironment triggering differentiation of classical monocytes into non-classical, macrophages and dendritic cells by mimicking the LPS effect (46). The reason why we did not see a similar IL-10 levels and monocytes relation in ER+ BC even with the same PLX effect (Figures 4A, 5A) may suggest that severity of the cancer and progesterone and ER play a role in plasma IL-10 levels, but this information is not elucidated in this study (47).

Higher IL-10 plasma levels were associated to poorer NAC outcomes (Table 2). Also, the percentage of classical monocytes was the highest in a patient with partial responses (Table 3). Despite this, when we compared immune markers vs. pCR differences did not reach statistical significance. Our conclusions are limited by the heterogeneity, the size of our sample and absence of a molecular definition for BC subtypes. However, our results show a trend that could guide future studies. Therefore, our findings should be further confirmed in larger studies increasing the number of patients and selecting only HER2-enriched tumors in order to perform more specific measurements of IL-10 secretion and classical monocytes, to demonstrate a consistent relationship.

## Conclusion

Our results suggest that peripheral blood monocyte subsets and plasma IL-10 levels in BC patients under NAC may serve as useful indicators to guide BC treatments in the future.

## Data Availability Statement

The datasets generated for this study are avasilable on request to the corresponding author.

## Ethics Statement

This study was approved by the Scientific Ethics committee of our institution (CEC-MEDUC); project 16-312 and follows the principles outlined by the Helsinki Declaration. The patients/participants provided their written informed consent to participate in this study.

## Author Contributions

AP-S designed the experiments and collected the clinical data with SM. JV-F and NM-D designed and performed the experiments and analyzed the obtained data. SM supported with the collection of the patient's samples and the logistics. IC-A design and performed the pCR statistics analyzes. JV-F lead the wrote of the manuscript with the support of all the authors. SB, JS, and FA helped to write and edit the manuscript. AK and CS gave direction and provided financial support. All authors contributed to the article and approved the submitted version.

## Conflict of Interest

The authors declare that the research was conducted in the absence of any commercial or financial relationships that could be construed as a potential conflict of interest.

## Abbreviations

BC: Breast Cancer
NAC: Neoadjuvant Chemotherapy
HER2: Human epidermal growth factor receptor type 2
ALC: Absolute lymphocyte count
AMC: Absolute monocyte count
ANC: Absolute neutrophil count
ER: Estrogen receptor
TN: Triple negative
PBMC: Peripheral blood mononuclear cells
MDSC: Myeloid derived suppressor cells
NLR: Neutrophil to Lymphocyte Ratio
PLX: Paclitaxel
DOX: Doxorubicine
pCR: Pathologic complete response.
