# Supplementary material for: Peripheral Blood Classical Monocytes and Plasma Interleukin 10 Are Associated to Neoadjuvant Chemotherapy Response in Breast Cancer Patients
Source: Front Immunol. 2020 Jul 9;11:1413. doi: 10.3389/fimmu.2020.01413 (PMC7363840; doi:10.3389/fimmu.2020.01413)
Supplement: Supplementary file 1 [file Data_Sheet_1.docx]

**SUPPLEMANTARY DATA (Additional files)**


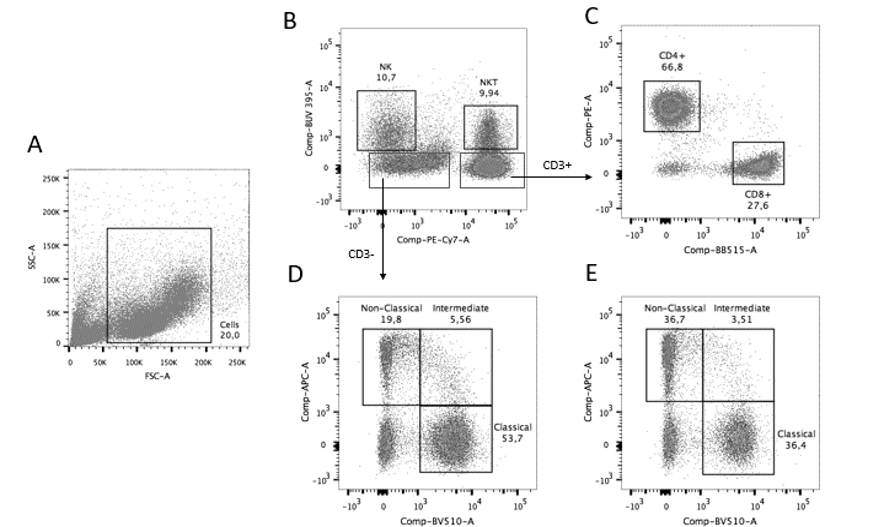


**Additional file 1: Figure S1. Gaiting strategy flow cytometry to evaluate Lymphocyte, Monocytes and Natural Killer cells populations in PBMCs.** PBMCs were analyzed by flow cytometry to evaluate the presence of CD4^+^ T cells, CD8^+^T cells, natural killer cells and monocytes subsets. In A) the total of cells and the arrows shows the gated population. B) the natural killer cells (CD3^Neg^CD56^+^) and NKT cells in C) The CD4^+^ T cells (CD3^+^CD4^+^) with CD8 + T Cells (CD3^+^ CD8^+^) and in D) and E) we identified the monocytes populations in different cycles for the same patient. The markers for each monocyte was: Classical monocytes (CD14^High^CD16^Neg^) Intermediate (CD14^+^CD16^+^) and Non-Classical monocytes (CD14^+^CD16^High^).

**Additional file 2: Figure 2. TGFβ and TNFα levels in plasma from breast cancer patient.** The cytokines were measured in plasma samples from breast cancer patients across the NAC cycles (n = 20). For both cytokines the cycle 1 (C1) is the effect of the treatment without any drug (baseline). In (a) the effect of the doxorubicin (DOX) for TGFβ and in (b) for paclitaxel (PLX). Also, in (c) the effect of DOX and in (d) the effect of PLX for the TNFα plasma levels. All data presented as optical density units (OD) with +/- SEM for each NAC cycle. Statistical significances were assessed using a One-way ANOVA test *p < 0.05.
